# Liproxstatin-1 improves boar sperm quality during *in vitro* liquid preservation at 17°C, associated with reduction of oxidative stress and ferroptosis markers

**DOI:** 10.3389/fvets.2025.1611661

**Published:** 2025-07-31

**Authors:** Yang Li, Xue Liu, Ye Cheng, Jingchun Li, Yuling Zhou, Qing Guo

**Affiliations:** ^1^College of Animal Science and Technology, Heilongjiang Bayi Agricultural University, Daqing, China; ^2^Hangzhou Dimoman Biotechnology, Hangzhou, China

**Keywords:** boar semen liquid preservation, Liproxstatin-1, sperm quality, ferroptosis, erastin

## Abstract

**Objective:**

The plasma membrane of boar sperm is notably enriched in polyunsaturated fatty acids (PUFAs). During extended liquid storage of boar semen at 17°C, reactive oxygen species (ROS) derived from lipid peroxidation progressively accumulate within sperm cells. Concurrently, the onset of ferroptosis is initiated by the disruption of intracellular redox homeostasis, characterized by an imbalance between the production and elimination of lipid-derived ROS. This study aims to investigate whether the ferroptosis inhibitor Liproxstatin-1 (Lip-1) protects boar sperm quality during 17°C liquid preservation by ameliorating oxidative stress and regulating ferroptosis markers.

**Method:**

Various concentrations of Lip-1 were added to the modified Modena extender, and sperm motility and kinetic parameters were assessed using the CASA system, which facilitated the identification of the optimal Lip-1 concentration. Subsequently, the integrity of the acrosome, plasma membrane, and mitochondrial membrane potential (MMP) of sperm was examined in both the control group and the optimal of Lip-1 group. Additionally, the antioxidant capacity and lipid peroxidation levels of the sperm were evaluated. Furthermore, the ferroptosis inducer Erastin (Era) was utilized to investigate whether Lip-1 could regulate oxidative stress and ferroptosis markers to enhance the liquid preservation efficiency of boar semen at 17°C.

**Result:**

Various concentrations of Lip-1 were added to the modified Modena extender, and the results indicated that, compared to the control group, 0.2 μM of Lip-1 significantly enhanced sperm motility and kinetic parameters. Additionally, a concentration of 0.2 μM Lip-1 significantly enhanced sperm quality, which included improvements in the integrity of the sperm plasma membrane and acrosome, antioxidant capacity, and MMP. Additional, additional tests revealed that Lip-1 can significantly reduce markers of sperm lipid peroxidation during the room temperature preservation of boar semen, including C11-bodipy, MDA, LPO, and improved ferroptosis-related protein GPX4. Furthermore, the ferroptosis inducer Era was utilized, and the results demonstrated that 0.2 μM Lip-1 significantly alleviated the sperm damage induced by Era.

**Conclusion:**

The results of this study indicated that Lip-1 significantly enhanced the liquid preservation efficiency of boar semen at 17°C associated with ameliorating oxidative stress and regulating ferroptosis markers, providing both theoretical and practical references for improving the liquid preservation of boar semen.

## 1 Introduction

Artificial insemination (AI) technology has been widely adopted globally, significantly enhancing the economic benefits of the porcine breeding industry (1). Boar semen is typically preserved in liquid at 17°C, primarily to suppress sperm metabolism and motility, thereby extending the duration of sperm preservation *in vitro* (2). However, as the preservation time increases, an imbalance between the antioxidant defense system and the production of reactive oxygen species (ROS) leads to oxidative stress, which results in a decline in sperm quality and fertilization capability (3). Therefore, it is crucial to explore effective strategies and methods to enhance the quality of sperm during semen liquid preservation at 17°C, as this is of great importance for the porcine breeding industry.

The plasma membrane of boar sperm contains a high concentration of polyunsaturated fatty acids (PUFAs), which are particularly sensitive to ROS, leading to the accumulation of lipid peroxides (4). Ferroptosis is characterized by the accumulation of lipid peroxides and the depletion of the intracellular antioxidant glutathione peroxidase 4 (GPX4), resulting in damage to the cell membrane and ultimately cell death (5). Furthermore, the sperm plasma membrane is progressively compromised due to the assault of ROS and the accumulation of lipid peroxides, which is a principal factor contributing to the gradual decline in sperm quality and fertilization ability during liquid preservation at 17°C (6, 7). As the sperm plasma membrane sustains damage, iron ions within the sperm may be released. However, the dysregulation of iron leads to a reduction in GPX4, a key regulator of ferroptosis (8), which is associated with various diseases, including Alzheimer’s disease (9). An amino acid antiporter, composed of the Slc7A11 and Slc3A2 subunits, is embedded in a phospholipid bilayer of the cell membrane. This antiporter facilitates the transport of cystine into the cell, where it is subsequently reduced to cysteine, thereby contributing to the synthesis of glutathione (GSH) (10). Disruption of this transport impedes cysteine synthesis within cells, resulting in decreased levels of GSH and, consequently, a reduction in GPX4 levels. The inactivation of GPX4 results in excessive accumulation of lipid ROS, which causes lipid peroxidation damage and induces ferroptosis in cells (8). Therefore, during the liquid preservation process of boar semen, ferroptosis may occur as preservation time extends, ultimately leading to a decline in sperm quality.

Liproxstatin 1 (Lip-1) is a selective inhibitor of ferroptosis, effectively preventing this form of cell death across various cells and tissues by inhibiting lipid peroxidation (11). Moreover, Lip-1 can completely inhibit lipid peroxidation in GPX4^−/−^ cells and modulate the expression of key ferroptosis-related marker proteins, specifically GPX4 and Slc7A11, thereby preventing ferroptosis (12). Therefore, this study aims to investigate whether the ferroptosis inhibitor Lip-1 protects boar sperm quality during 17°C liquid preservation by ameliorating oxidative stress and regulating ferroptosis markers. The results of this study will provide both theoretical and practical references for enhancing the liquid preservation efficiency of boar semen within the porcine breeding industry.

## 2 Methods

### 2.1 Chemicals

Lip-1 (>98% HPLC) was obtained from Chengdu Preferred Biotechnology Co., Ltd. (Chengdu, China), while the kits for malondialdehyde (MDA), lipid peroxidation (LPO), GSH, and total antioxidant capacity (T-AOC) were sourced from Nanjing Jiancheng Bioengineering Institute (Nanjing, China). Unless otherwise stated, all chemicals used in this study were purchased from Sigma-Aldrich (St. Louis, MO, USA). The modified Modena extender contains 46.64 mM Tris, 152.64 mM glucose, 15.09 mM citric acid, 26.74 mM sodium citrate, 6.98 mM EDTA-2Na·H_2_O, 11.90 mM sodium bicarbonate, 1 million U/L penicillin, 1 million U/L streptomycin, and 4.00 g/L BSA, which is prepared prior to use and stored at 4°C.

### 2.2 Semen collection and treatment

In this experiment, six Large White boars aged 2 to 3 years were utilized. The boars were individually housed at a controlled temperature of 20 ± 2°C and were provided unrestricted access to food (Shenyang Hefeng Animal Husbandry, Shenyang, China) and water, ensuring no impact on the quality of boar semen. For each experiment, semen was collected from a boar using the gloved-hand method, and sperm motility was subsequently analyzed using the Computer Assisted Semen Analysis (CASA) system (Songjingtianlun Biotechnology, Nanning, China). The samples were transported to the laboratory within 2 h and were utilized for subsequent experiments only when sperm total motility exceeded 80%. Initially, various concentrations of Lip-1 were added to the modified Modena extender of boar semen, which was maintained in liquid form at 17°C. Sperm motility was assessed on days 1, 3, 5, and 7 of preservation to determine the optimal concentration of Lip-1. Additionally, sperm quality was evaluated at 0, 1, 3, 5, and 7 days, measuring parameters such as the integrity of the acrosome and plasma membrane, and mitochondrial membrane potential (MMP). The assessment included measures of antioxidant capacity, such as GSH and T-AOC; Lipid peroxidation index MDA, LPO, and ferroptosis indicators C11-bodipy. Additionally, we utilized the ferroptosis inducer erastin (Era, 1 μM) to investigate the mechanism by which Lip-1 enhances boar semen fluid preservation efficiency at 17°C.

### 2.3 Measurement of sperm motility and kinetic parameters

Transfer 10 μL of the sample onto a glass slide and cover it with a coverslip. Analyze the motility and kinetic parameters of the sperm using the CASA system, which includes total motility, progressive motility, average path velocity (VAP), average straight-line velocity (VSL), and average curvilinear velocity (VCL). The standard parameter for CASA was set to 30 frames/s. Sperm with a straightness of path (STR) greater than 75% and VSL exceeding 25 μm/s were classified as motile. Each sample was randomly selected across five fields, with a minimum of 200 sperm measured using the accompanying software.

### 2.4 Measurement of sperm MMP

MMP was detected using JC-1 and propidium iodide (PI) as in the previous study with appropriate modifications (13). The JC-1 probe aggregates in the mitochondrial matrix to form a polymer when the MMP is high (hMMP), while it is excluded from the mitochondrial matrix in cases of low MMP. Total 50 μL sample was mixed with 200 μL of an isotonic buffer diluent containing 5 mM PI and 1 mM JC-1, and the mixture was incubated for 30 min at 37°C. Subsequently, a 10 μL aliquot of the semen sample was placed onto a coverslip with a glass slide and observed using an inverted fluorescence microscope (Mshot Optoelectronics Technology, Guangzhou, China), with analysis performed using the associated software. More than 200 sperm were examined per visual field. Red fluorescence on the head of the sperm indicates hMMP, while green fluorescence represents medium to low MMP.

### 2.5 Measurement of sperm acrosome

The integrity of the sperm acrosome (14) was analyzed according to previous studies with appropriate modifications. 30 μL of the sample was placed on a slide, allowed to air dry, and then fixed with methanol for 10 min at 23°C. Each slide was treated with FITC-PNA solution (100 μg/mL) and incubated at 37°C for 10 min in the dark. After incubation, the slides were rinsed with phosphate-buffered saline (PBS) and mounted with an antifade solution to prevent fluorescence degradation. Finally, the slides were covered with a coverslip and photographed using an epifluorescence microscope to evaluate acrosome integrity.

### 2.6 Measurement of sperm plasma membrane integrity

Sperm plasma membrane staining method was adapted from a previous study with appropriate modifications (15). A 250 μL sample was centrifuged at 1200 r/min for 2 min. The supernatant was discarded, and 500 μL of HEPES buffer containing 10% BSA was added. Subsequently, 5 μL SYBR-14 dye working solution (0.1 mmol/mL) was added and incubated at 37°C for 5 min, followed by the addition of 5 μL of PI working solution (0.1 mg/mL) and incubation at 37°C for an additional 5 to 10 min. Observations were conducted using a fluorescence microscope in a dark room, examining 5 fields of view per group, with each field containing no fewer than 200 sperm.

### 2.7 Measurement of sperm LPO, MDA, GSH, and T-AOC

LPO content, MDA content, GSH content, and T-AOC activity in sperm were assessed following the kit instructions and measured using a microplate reader. The parameters for the microplate reader were set to 450 nm, 532 nm, 412 nm, and 593 nm, respectively.

### 2.8 Measurement of sperm C11-bodipy level

The semen samples from each treatment group were centrifuged and subsequently re-suspended in 500 μL of C11-bodipy working solution (1 μM), followed by incubation at 37°C in the dark for 1 h. After incubation, the semen samples were centrifuged again and washed three times with PBS. The stained sperm samples were re-suspended in PBS to achieve a density of 10^6^ sperm/mL and analyzed using a flow cytometer (Beckman Coulter, Brea, USA). The fluorescence signal intensity of the sperm was assessed through the red fluorescence channel at 488/510 nm, with a total of 10,000 sperm detected for each sample.

### 2.9 Immunofluorescence analysis of GPX4 protein expression in boar sperm

The expression of GPX4 protein in boar sperm was analyzed using immunofluorescence staining. Semen samples were washed three times with PBS, and the sperm density was adjusted to 10^6^/mL. A 30 μL aliquot of the semen sample suspension was prepared for smearing, fixed in 4% paraformaldehyde for 10 min, and subsequently permeabilized with 0.5% Triton® X-100 for 30 min. Following three washes with PBS, 200 μL of 5% BSA was added to block non-specific binding, and the samples were incubated at 37°C for 30 min. Subsequently, the samples were centrifuged, and the supernatant was carefully removed. Meanwhile, 200 μL of diluted GPX4 antibodies (1:1000) were added and incubated at 4°C overnight. Afterward, the relevant fluorescent secondary antibodies (1:1000) were applied for incubation at 37°C for 1 h, and sperm cell nuclei were stained with 4′,6-diamidine-2-phenylindole dihydrochloride (DAPI). The slides were sealed, and images were captured using a fluorescence microscope (Mshot Photoelectric Technology, Guangzhou, China), with data digitization performed using iMage software (Olympus, Tokyo, Japan).

### 2.10 Statistical analysis

Statistical analysis of experimental data was performed using SPSS 17.0. All data from each experiment were tested for normality with one-sample Kolmogorov–Smirnov’s test. If some data did not show normality, we arcsine-transformed the variable before the analysis and again checked the normality using one sample Kolmogorove-Smirnov’s test for this parameter. Furthermore, all data from each experiment were checked for the homogeneity of variances using Levene’s test. The test results showed that the replicated data from each experiment were homogeneous. The values are presented as mean ± standard deviation (SD), and multiple comparisons were analyzed using one-way ANOVA followed by Tukey’s multiple-comparison test. The differences between the two groups of data were assessed using an unpaired two-tailed Student t-test. Significant differences are represented by *p* < 0.05.

## 3 Results

### 3.1 Lip-1 improved sperm motility and kinetic parameters

To investigate the protective effect of different concentrations Lip-1 on sperm damage during the liquid preservation of boar semen at 17°C, we examined sperm motility and kinetic parameters. The results indicated that, in comparison to the control group, treatments with 0.1 μM and 0.4 μM Lip-1 did not produce significantly effects on sperm total motility on days from 1 to 7 (Table 1, *p* > 0.05). In contrast, treatment with 0.2 μM Lip-1 significantly improved sperm total motility compared to the control group from days 3 to 7 (Table 1, *p* < 0.05). Additionally, 0.1 μM Lip-1 significantly improved the progressive motility of sperm relative to the control group on day 1 (Table 2, *p* < 0.05), it did not significantly affect sperm progressive motility on days from 3 to 7 (Table 2, *p* > 0.05). Meanwhile, compared to the control group, 0.4 μM Lip-1 showed no significant effects on sperm progressive motility on days from 1 to 7 day (Table 2, *p* > 0.05). In contrast, 0.2 μM Lip-1 significantly improved the sperm progressive motility from 1 to 7 day in comparison to the control group (Table 2, *p* < 0.05).

**Table 1:** Effects of different concentrations of Lip-1 on boar spermatozoa total motility (%).

Groups	Storage time (day)			
	1	3	5	7
Control	94.37 ± 0.44^a^	84.00 ± 0.60^b^	70.45 ± 0.87^b^	48.75 ± 0.64^bc^
0.1 μM	93.46 ± 0.46^a^	83.70 ± 1.26^ab^	71.43 ± 0.29^b^	51.03 ± 0.90^b^
0.2 μM	93.85 ± 0.51^a^	87.68 ± 0.79^a^	76.62 ± 0.50^a^	57.19 ± 0.78^a^
0.4 μM	92.79 ± 0.33^a^	82.42 ± 1.03^b^	70.33 ± 2.14^ab^	46.78 ± 0.69^c^
*p* value	*p* > 0.05	*p* < 0.05	*p* < 0.05	*p* < 0.05

In the same column, values with different letter superscripts^(a-c)^ mean a significant difference (*p* < 0.05) on the same processing day, *n* = 3.

**Table 2:** Effects of different concentrations of Lip-1 on boar spermatozoa progressive motility (%).

Groups	Storage time (day)			
	1	3	5	7
Control	78.06 ± 1.23^b^	71.44 ± 0.78^b^	58.00 ± 0.74^b^	38.13 ± 1.36^b^
0.1 μM	88.33 ± 1.87^a^	71.43 ± 0.81^b^	59.81 ± 1.14^b^	41.59 ± 1.90^ab^
0.2 μM	89.01 ± 0.96^a^	75.90 ± 1.23^a^	65.05 ± 1.09^a^	46.83 ± 1.30^a^
0.4 μM	75.61 ± 0.73^b^	69.90 ± 0.25^b^	59.03 ± 0.75^b^	35.12 ± 0.84^b^
*p* value	*p* < 0.05	*p* < 0.05	*p* < 0.05	*p* < 0.05

In the same column, values with different letter superscripts^(a, b)^ mean a significant difference (*p* < 0.05) on the same processing day, *n* = 3.

For sperm kinetic parameters, on day 1, 0.2 μM Lip-1 significantly increased sperm VAP (Table 3, *p* < 0.05) compared to the control group, while 0.1, 0.2 and 0.4 μM Lip-1 Lip significantly enhanced sperm VSL (Table 4, *p* < 0.05) and VCL (Table 5, *p* < 0.05). Furthermore, on day 3, concentrations of 0.1 μM and 0.4 μM Lip-1 did not demonstrate any significant effects on sperm VAP (Table 3, *p* > 0.05), VSL (Table 4, *p* > 0.05), and VCL (Table 5, *p* > 0.05); a concentration of 0.2 μM Lip-1 significantly improved all three parameters (Tables 3–5, *p* < 0.05). Moreover, on day 5, 0.2 μM Lip-1 significantly increased sperm VAP (Table 3, *p* < 0.05), VSL (Table 4, *p* < 0.05) and VCL (Table 5, *p* < 0.05). Importantly, on day 7, both 0.1 μM and 0.2 μM concentrations of Lip-1 significantly increased sperm VAP (Table 3, *p* < 0.05). Additionally, 0.2 μM Lip-1 notably enhanced sperm VSL (Table 4, *p* < 0.05), while both 0.2 μM and 0.4 μM concentrations of Lip-1 significantly improved sperm VCL (Table 5, *p* < 0.05). In summary, a concentration of 0.2 μM Lip-1 significantly enhances sperm motility and kinetic parameters during the liquid storage of pig semen at 17°C. Consequently, 0.2 μM Lip-1 was chosen for subsequent experiments.

**Table 3:** Effects of different concentrations of Lip-1 on boar spermatozoa VAP (μm/s).

Groups	Storage time (day)			
	1	3	5	7
Control	58.66 ± 0.36^b^	48.12 ± 1.72^c^	50.53 ± 1.23^b^	38.72 ± 1.50^c^
0.1 μM	65.04 ± 1.00^b^	55.22 ± 0.44^b^	54.53 ± 2.78^ab^	44.54 ± 0.71^ab^
0.2 μM	73.47 ± 2.52^a^	59.14 ± 0.66^a^	55.08 ± 0.73^a^	51.28 ± 2.01^a^
0.4 μM	64.46 ± 0.98^b^	49.50 ± 0.51^c^	55.99 ± 2.60^ab^	43.23 ± 1.10^bc^
*p* valuse	*p* < 0.05	*p* < 0.05	*p* < 0.05	*p* < 0.05

In the same column, values with different letter superscripts^(a-c)^ mean a significant difference (*p* < 0.05) on the same processing day, *n* = 3.

**Table 4:** Effects of different concentrations of Lip-1 on boar spermatozoa VSL (μm/s).

Groups	Storage time (day)			
	1	3	5	7
Control	47.70 ± 1.60^c^	52.49 ± 0.77^b^	48.92 ± 2.08^bc^	44.89 ± 1.92^b^
0.1 μM	57.78 ± 2.41^ab^	56.96 ± 1.35^ab^	50.50 ± 0.72^b^	45.07 ± 3.21^ab^
0.2 μM	60.29 ± 1.22^a^	59.46 ± 1.02^a^	57.21 ± 1.74^a^	52.72 ± 0.64^a^
0.4 μM	55.51 ± 0.60^b^	56.80 ± 2.52^ab^	42.35 ± 1.52^c^	48.65 ± 1.65^ab^
*p* value	*p* < 0.05	*p* < 0.05	*p* < 0.05	*p* < 0.05

In the same column, values with different letter superscripts ^(a-c)^ mean a significant difference (*p* < 0.05) on the same processing day, *n* = 3.

**Table 5:** Effects of different concentrations of Lip-1 on boar spermatozoa VCL (μm/s).

Groups	Storage time (day)			
	1	3	5	7
Control	77.59 ± 1.31^c^	63.14 ± 3.14^b^	67.52 ± 0.52^b^	54.71 ± 1.14^b^
0.1 μM	85.97 ± 1.90^b^	74.00 ± 1.10^b^	72.53 ± 2.34^b^	53.77 ± 3.56^ab^
0.2 μM	98.05 ± 1.87^a^	80.58 ± 3.38^a^	75.26 ± 0.75^a^	60.92 ± 0.89^a^
0.4 μM	89.61 ± 1.02^b^	70.50 ± 0.47^b^	65.42 ± 3.70^b^	59.87 ± 0.43^a^
*p* value	*p* < 0.05	*p* < 0.05	*p* < 0.05	*p* < 0.05

In the same column, values with different letter superscripts^(a-c)^ mean a significant difference (*p* < 0.05) on the same processing day, *n* = 3.

### 3.2 Lip-1 improved sperm acrosome integrity and plasma membrane integrity

**Figure 1:**
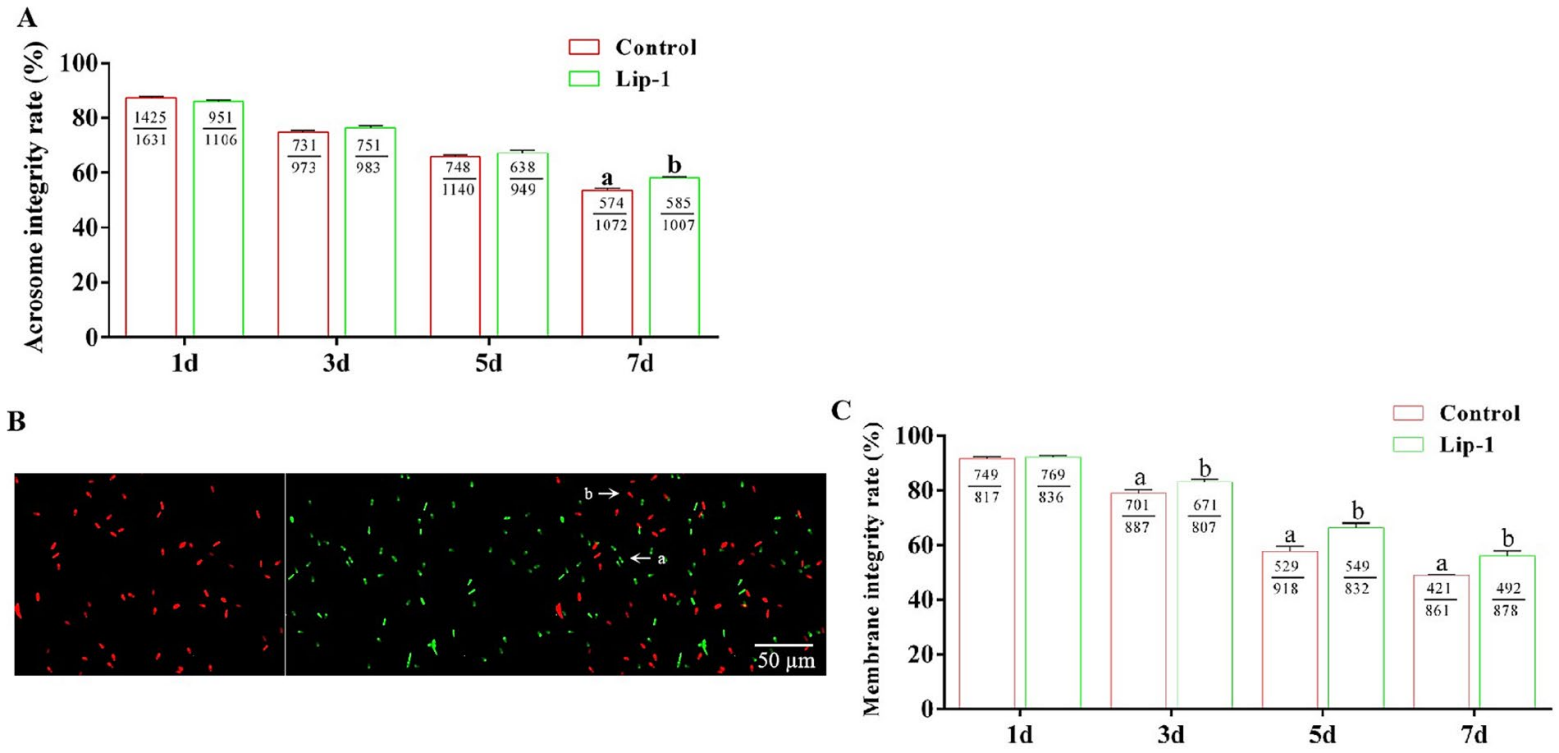
Effects of Lip-1 on the acrosome and plasma membrane integrity of boar sperm. **(A)** Sperm acrosome intact rate (%). **(B)** SYBR-14/PI staining for sperm plasma membrane integrity. Among them, a indicates intact sperm plasma membrane, whereas b denotes sperm plasma membrane damage. Scale bars: 50 μm. **(C)** Sperm plasma membrane intact rate (%). *n* = 3, Data are demonstrated as means ± SD, different letters indicate significant difference (*p* < 0.05) on the same processing day.

The integrity of the acrosome in spermatozoa is a critical factor for the occurrence of the acrosomal reaction during fertilization and for the successful penetration of sperm into oocytes (16, 17). Consequently, we investigated the effect of 0.2 μM Lip-1 on the acrosomal integrity of sperm during liquid storage at 17°C. The result showed that, on days 1, 3, and 5, treatment with 0.2 μM Lip-1 did not significantly (Figure 1A, *p* > 0.05) effect the acrosome integrity of sperm compared to the control group; however, on day 7, it significantly enhanced the acrosome integrity (Figure 1A, *p* < 0.05).

Additionally, the integrity of the sperm plasma membrane serves as a crucial barrier between sperm cells and the external environment, significantly influencing several key reproductive processes, including sperm survival, capacitation, and acrosomal reaction (18). In this study, we utilized SYBR-14/PI staining to assess the integrity of the sperm cell membrane. Specifically, ‘a’ denotes intact sperm cell membranes, whereas ‘b’ signifies damaged sperm cell membranes (Figure 1B). The result showed that, on day 1, relative to the control group, treatment with 0.2 μM Lip-1 showed no significant (Figure 1C, *p* > 0.05) effect on plasma membrane integrity; however, on days 3, 5, and 7, it significantly (Figure 1C, *p* < 0.05) improved this effect.

### 3.3 Lip-1 improved sperm hMMP

**Figure 2:**
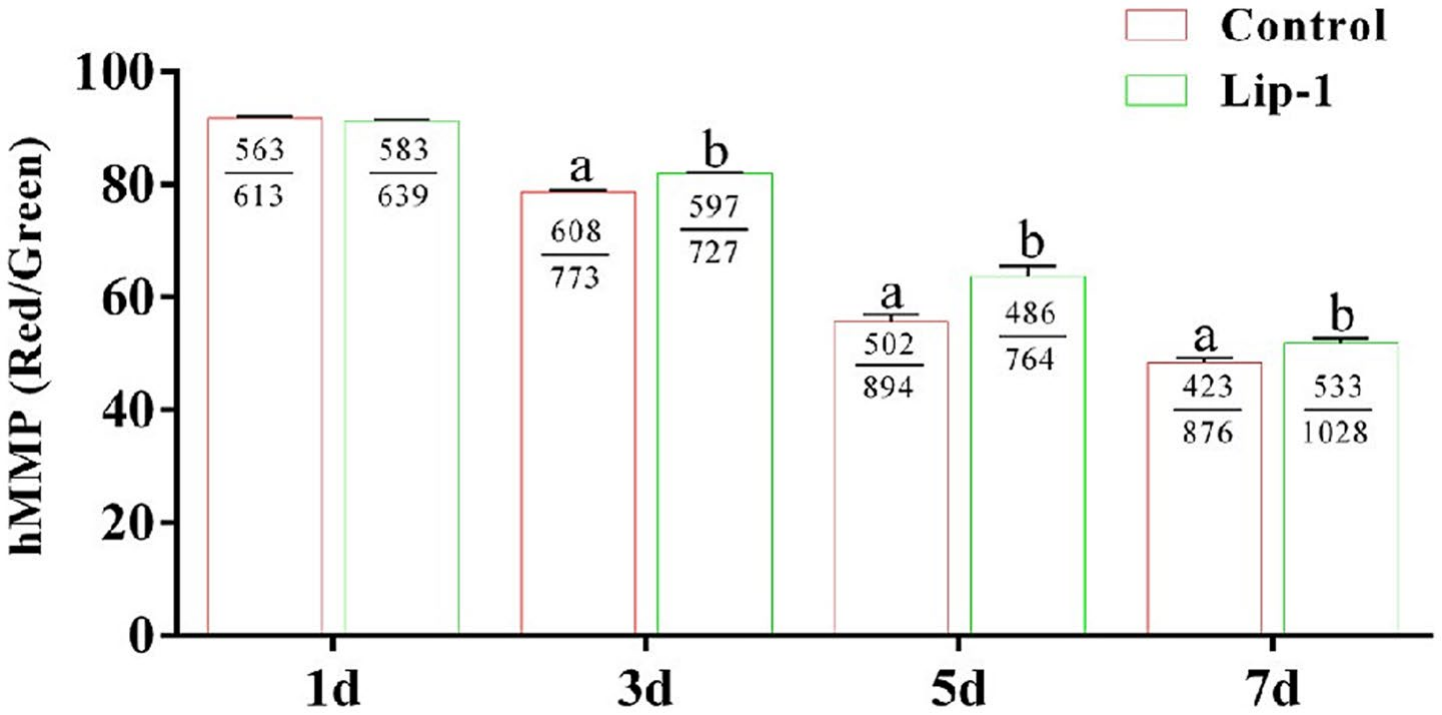
Effects of Lip-1 on boar sperm MMP level. *n* = 3, Data are demonstrated as means ± SD, different letters indicate significant difference (*p* < 0.05) on the same processing day.

Sperm with low MMP demonstrate a reduced capacity for ATP production, whereas those with hMMP are capable of generating greater amounts of ATP to facilitate sperm motility (19, 20). Consequently, we investigated the effect of 0.2 μM Lip-1 on the MMP of sperm. The result showed that, on day 1, compared to the control group, treatment with 0.2 μM Lip-1 had no significant (Figure 2, *p* > 0.05) effect on the hMMP of sperm; nonetheless, on days 3, 5, and 7, it significantly (Figure 2, *p* < 0.05) enhanced the hMMP.

### 3.4 Lip-1 improved sperm antioxidant capacity

**Figure 3:**
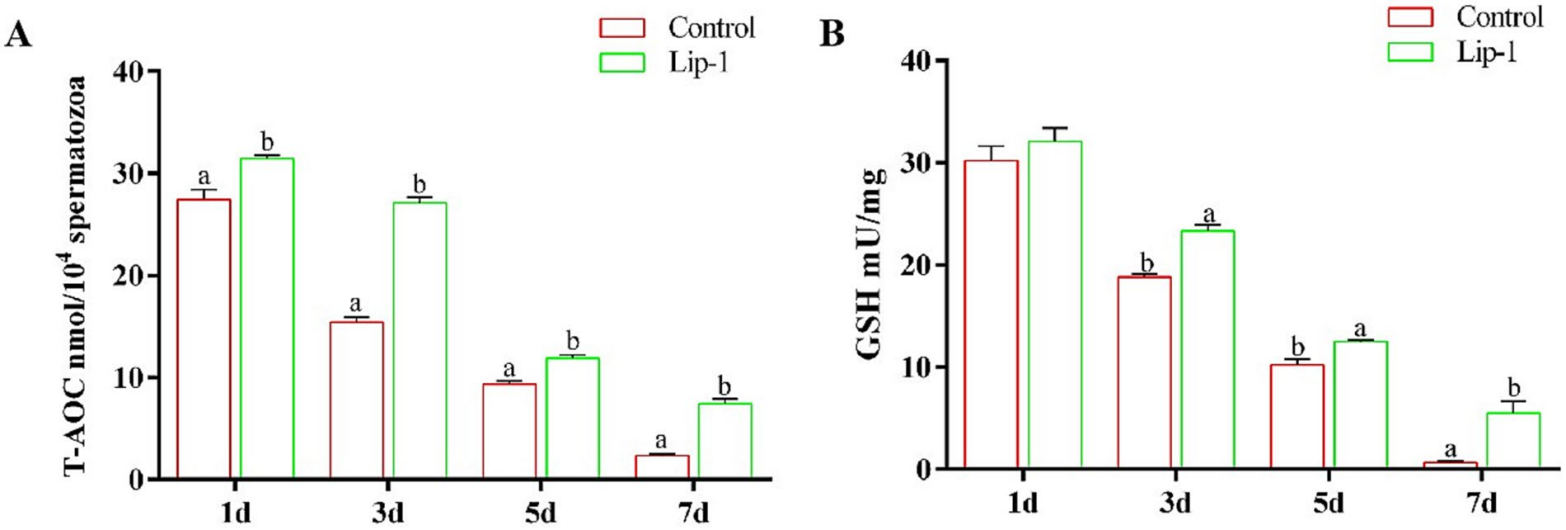
Effects of Lip-1 on boar sperm antioxidant capacity. **(A)** T-AOC activity, **(B)** GSH contents. *n* = 3, Data are demonstrated as means ± SD, different letters indicate significant difference (*p* < 0.05) on the same processing day.

During the liquid preservation of boar semen at room temperature, oxidative stress emerges as a significant factor contributing to sperm damage (6). Consequently, we measured the T-AOC activity and GSH content in sperm during liquid preservation. The results indicated that, on days from 1 to 7, compared to the control group, 0.2 μM Lip-1 significantly increased the activity of T-AOC (Figure 3A, *p* < 0.05) in sperm. Meanwhile, on days from 3 to 7, the content of GSH in 0.2 μM Lip-1 group was significantly higher than that of control group (Figure 3B, *p* < 0.05).

### 3.5 Lip-1 decreased sperm LPO and MDA contents, and C11-bodipy level and improved GPX4 protein

**Figure 4:**
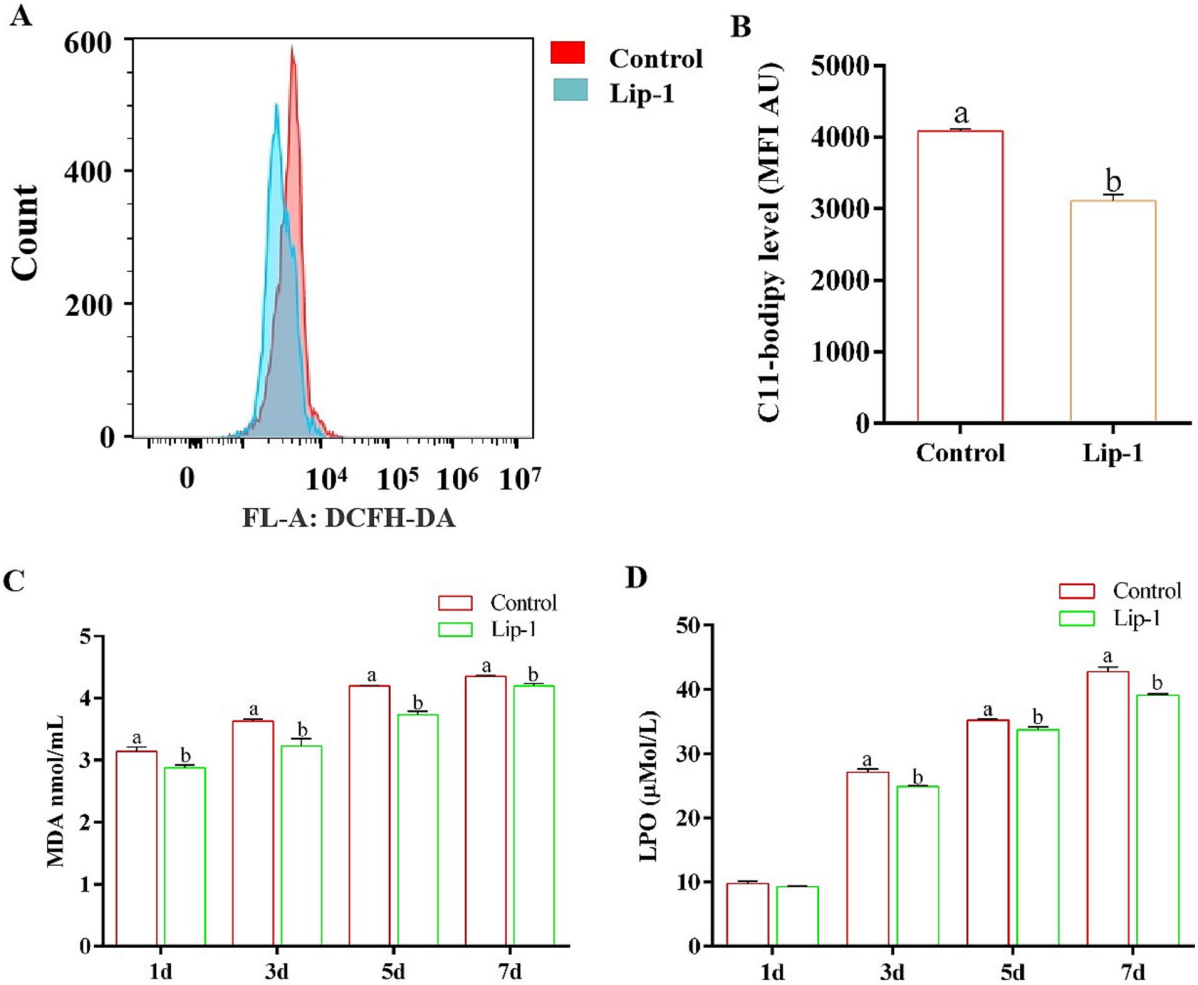
Effects of Lip-1 on boar sperm ferroptosis marker including C11-bodipy levels, MDA, and LPO contents. **(A)** Flow cytometry detected sperm C11-bodipy levels at day 5. **(B)** Sperm C11-bodipy levels. **(C)** MDA contents at days 1, 3, 5, 7. **(D)** LPO contents at days 1, 3, 5, 7. *n* = 3, Data are demonstrated as means ± SD, different letters indicate significant difference (*p* < 0.05) on the same processing day.

**Figure 5:**
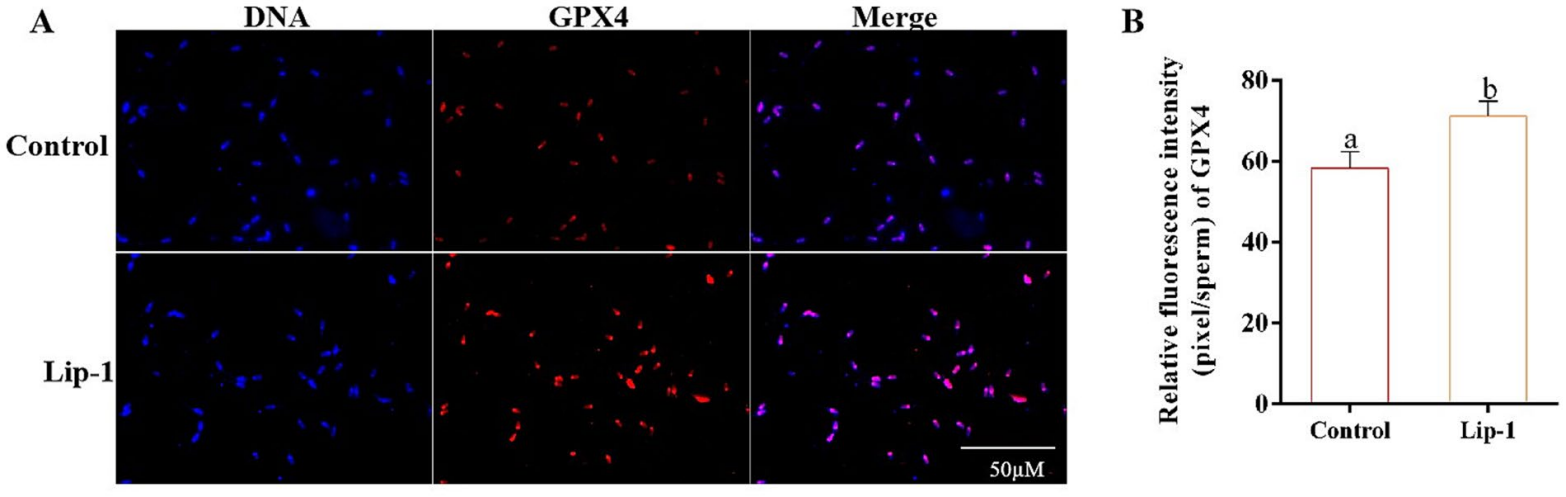
Effects of Lip-1 on boar sperm ferroptosis-related proteins GPX4. **(A)** Immunofluorescence staining for GPX4 proteins. **(B)** Data analysis of GPX4 protein. *n* = 3, Data are demonstrated as means ± SD, different letters indicate significant difference (*p* < 0.05) on the same processing day.

Since Lip-1 is a ferroptosis inhibitor that can protect cells by regulating lipid peroxidation (11), we examined lipid peroxide markers, specifically C11-bodipy, LPO, and MDA. The results showed that, compared to the control group, treatment with 0.2 μM Lip-1 significantly inhibited the levels of C11-bodipy in sperm on day 5 (Figures 4A,B, *p* < 0.05). Additionally, on days from 1 to 7, Lip-1 significantly reduced the content of MDA in sperm compared to the control group (Figure 4C, *p* < 0.05). Moreover, on days 3, 5, and 7, Lip-1 significantly decreased the content of LPO (Figure 4D, *p* < 0.05) in sperm relative to the control group. Furthermore, we examined ferroptosis-related protein GPX4 in sperm. The results indicated that, compared to the control group, Lip-1 significantly enhanced GPX4 protein levels in boar sperm during *in vitro* liquid preservation at room temperature on day 5 (Figure 5, *p* < 0.05).

### 3.6 Lip-1 improved the sperm motility and kinetic parameters, acrosome, plasma membrane, and MMP in era-treated sperm

To confirm that Lip-1 enhances boar sperm quality by regulating ferroptosis, we utilized the ferroptosis inducer, Era, to assess the protective effect of Lip-1 against Era-induced injury in boar sperm. The results showed that Era significantly decreased sperm total motility (Table 6, *p* < 0.05), progressive motility (Table 6, *p* < 0.05), VAP (Table 6, *p* < 0.05), VSL (Table 6, *p* < 0.05), VCL (Table 6, *p* < 0.05), and BCF (Table 6, *p* < 0.05) compared to the control group, but Lip-1 was significantly (Table 6, *p* < 0.05) recovered these changes.

**Table 6:** Effects of Lip-1 on boar sperm motility and kinetic parameters in Era-treated sperm.

Groups	Total motility (%)	Progressive motility (%)	VAP/ (μm/s)	VSL/ (μm/s)	VCL/ (μm/s)	BCF/ (Hz)
Control	92.27 ± 0.24^a^	86.90 ± 2.22^a^	74.26 ± 1.17^a^	74.75 ± 2.14^a^	105.78 ± 1.67^a^	23.69 ± 1.00^a^
Era	82.58 ± 0.49^c^	72.22 ± 1.45^b^	61.80 ± 3.11^b^	63.24 ± 5.96^b^	87.95 ± 4.40^b^	13.20 ± 2.36^b^
Era+Lip-1	87.27 ± 0.46^b^	83.02 ± 0.44^a^	74.07 ± 1.15^a^	78.44 ± 3.43^a^	105.50 ± 1.64^a^	16.53 ± 1.40^b^
*p* value	*p* < 0.05	*p* < 0.05	*p* < 0.05	*p* < 0.05	*p* < 0.05	*p* < 0.05

In the same column, values with different letter superscripts^(a, b)^ mean a significant difference (*p* < 0.05) among the three groups: control, Era, and Era+Lip-1, *n* = 3.

**Figure 6:**
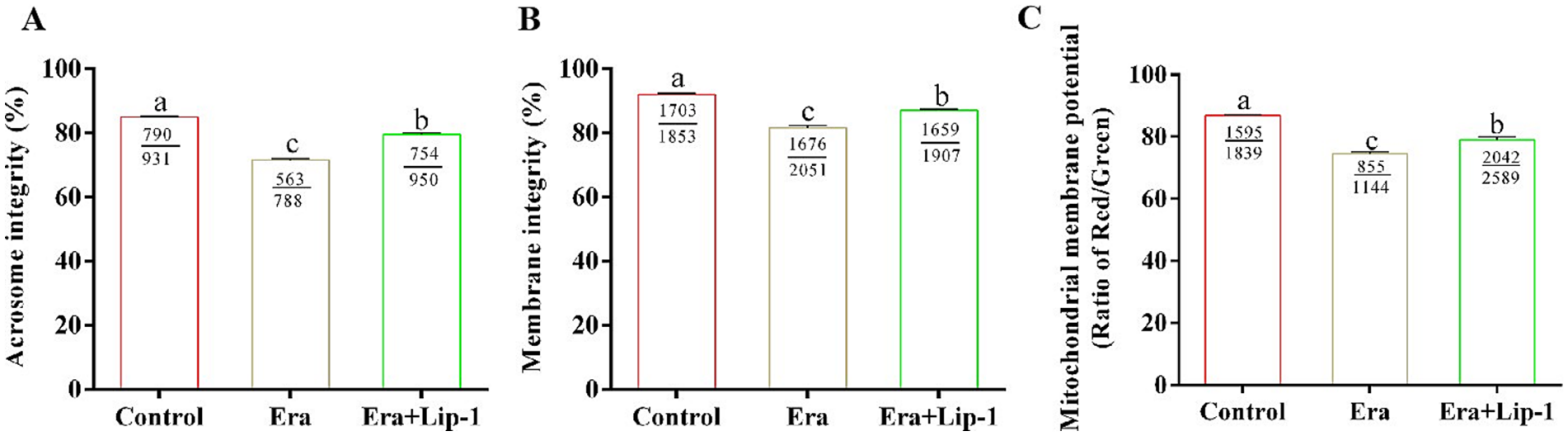
Effects of Lip-1 on acrosome and plasma membrane intact rate, and hMMP level in Era-treated sperm at 3 h. **(A)** Sperm acrosome integrity rate (%). **(B)** Sperm plasma membrane integrity rate (%). **(C)** Sperm hMMP level. *n* = 3, Data are demonstrated as means ± SD, different letters indicate significant difference (*p* < 0.05) among the three groups of control, Era and Era+Lip-1.

Moreover, Lip-1 improved the integrity of sperm acrosome and plasma membrane, and hMMP in Era-treated sperm as shown in Figure 6. In comparison to the control group, Era significantly reduced boar sperm quality including the integrity of acrosome (Figure 6A, *p* < 0.05) and plasma membrane (Figure 6B, *p* < 0.05), and MMP (Figure 6C, *p* < 0.05), whereas 0.2 μM Lip-1 significantly restored these damages (Figures 6A–C, *p* < 0.05).

### 3.7 Lip-1 decreased lipid peroxidation and improved antioxidant capacity in era-treated sperm

**Figure 7:**
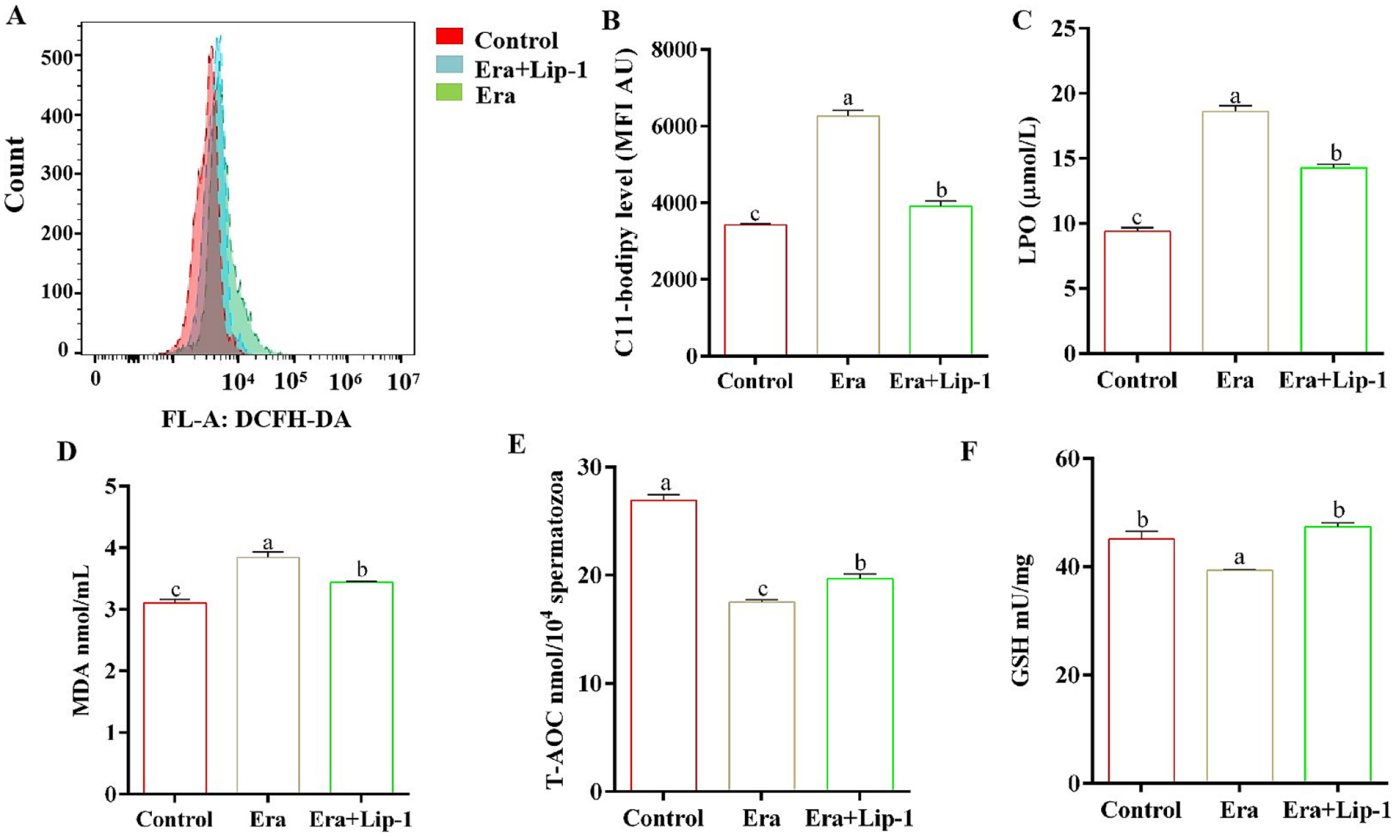
Effects of Lip-1 on sperm ferroptosis marker and antioxidant capacity in Era-treated sperm at 3 h. **(A)** Flow cytometry detected sperm C11-bodipy levels. **(B)** Sperm C11-bodipy levels. **(C)** LPO contents, **(D)** MDA contents, **(E)** T-AOC activity, **(F)** GSH contents. *n* = 3, Data are demonstrated as means ± SD, different letters indicate significant difference (*p* < 0.05), among the three groups: control, Era, and Era+Lip-1.

To further investigate the effect of Lip-1 on boar sperm quality by the regulation of ferroptosis, we assessed both the lipid peroxidation capacity and the antioxidant capacity of the sperm. The results demonstrated that Era significantly increased the levels of C11-bodipy (Figures 7A,B, *p* < 0.05), LPO (Figure 7C, *p* < 0.05), and MDA (Figure 7D, *p* < 0.05) compared with the control group, but 0.2 μM Lip-1 significantly restored these changes (Figures 7A–D, *p* < 0.05). Furthermore, in comparison to the control group, Era significantly reduced the antioxidant capacity of sperm, primarily including the activity of T-AOC (Figure 7E, *p* < 0.05) and the content of GSH (Figure 7F, *p* < 0.05), whereas the application of 0.2 μM Lip-1 notably ameliorated these alterations (Figures 7E,F, *p* < 0.05).

## 4 Discussion

Boar semen is primarily preserved in liquid form; however, time-dependent structural and biochemical impairments occur in sperm during the preservation at 17°C, leading to a decline in their fertilization capacity. Currently, various exogenous antioxidants were added to extender to reduce the levels of ROS by regulating lipid peroxidation. There strategy aims to alleviate the time-dependent structural and biochemical damage to sperm, thereby enhancing the efficiency of *in vitro* liquid preservation of boar semen (21–23). Meanwhile, the onset of ferroptosis is frequently associated with the accumulation of lipid peroxides and the depletion of GPX4, which leads to cell membrane damage and ultimately results in cell death (5). Numerous studies have demonstrated that Lip-1 can effectively inhibit ferroptosis by regulating lipid peroxidation (24–26). Consequently, this study focused on whether Lip-1 can improve the preservation efficiency of boar semen at room temperature by regulating ferroptosis.

The structural characteristics of mammalian sperm significantly influence their motility and kinetic parameters, which subsequently impacts the ability of the sperm to penetrate the egg (27). Therefore, during the liquid preservation of semen at 17°C, structural damage to sperm significantly reduces their fertilization capacity. Our results indicated that the sperm motility and kinetic parameters of the 0.2 μM Lip-1 samples were significantly higher than the other samples under *in vitro* liquid storage (17°C). Mitochondria are predominantly located in the tail of the sperm, with each mature sperm containing approximately 72 to 80 mitochondria (28). The ATP produced by these mitochondria is essential for maintaining the stability of the intracellular environment and providing energy for various physiological processes, particularly sperm motility (19, 29, 30). hMMP of mitochondrial indicates that sperm exhibit normal energy metabolism, where a hMMP is typically associated with high content of ATP (20). Our further detection revealed that 0.2 μM Lip-1 significantly increased the hMMP of sperm.

The integrity of the acrosome and plasma membrane is crucial for sperm fertilization capacity and serves as important indicators of sperm quality (28–30). Following the encounter between sperm and oocyte in the oviduct, sperm with intact acrosomes typically undergo the acrosome reaction, during which enzymes are released that penetrate the zona pellucida of the oocyte, thereby facilitating the completion of fertilization (31). Studies have demonstrated that reducing free radicals in sperm can mitigate oxidative damage to the sperm cell membrane, thereby better preserving the integrity of its structural composition (32). This study demonstrated that 0.2 μM Lip-1 significantly improves the integrity of both the sperm acrosome and plasma membrane.

Oxygen-free radical reactions and lipid peroxidation are critical components in body metabolic processes (33). Under normal circumstances, these two processes operate in a coordinated and dynamic equilibrium, facilitating numerous physiological and biochemical reactions (33). However, when this equilibrium is disrupted, it can lead to various metabolic disorders, initiating a cascade of oxygen free radical production and damaging biological membranes. Increased lipid peroxidation results in elevated levels of LPO, which can adversely affect on the structure and function of cells membranes (34). Moreover, MDA, one of the final products of lipid peroxidation in living organisms, serves as a significant indicator for assessing lipid peroxidation levels and oxidative stress (35). Additionally, the level of C11-bodipy significantly increases during the process of ferroptosis (36). Consequently, the contents of MDA, LPO, and C11-bodipy are frequently regarded as markers of ferroptosis. Conversely, in this study, we observed that during the liquid preservation of boar semen, the contents of MDA and LPO, as well as the level of C11-bodipy in the 0.2 μM Lip-1 group, were significantly lower than those in the control group.

Numerous studies have indicated that Lip-1 can inhibit ferroptosis in a variety of cells and tissues (11, 37, 38). However, we observed that Lip-1 significantly enhanced GPX4 protein levels in boar sperm during *in vitro* liquid preservation at room temperature on day 5, which suggests that Lip-1 may enhance the efficiency of room-temperature storage of porcine semen by mitigating ferroptosis. Numerous studies have demonstrated that Era can induce ferroptosis in various cell types, making it a widely used to constructing ferroptosis models (39–41). To further assess the effect of Lip-1 on the efficiency of boar semen liquid preservation by regulating ferroptosis, this study used the ferroptosis inducer Era to establish a boar sperm ferroptosis model. The results indicated that a concentration of 1 μM Era significantly decreased sperm total motility, kinetic parameters, the integrity of both the sperm acrosome and plasma membrane, MMP, and antioxidant capacity, while also improving lipid peroxidation levels. However, treatment with 0.2 μM Lip-1 significantly restored these alterations. These results further suggest that Lip-1 may enhance the efficiency of boar semen preservation at 17°C by regulating ferroptosis.

In conclusion, supplementation of Lip-1 at a concentration of 0.2 μM in modify extender significantly improved sperm motility and kinetic parameters during *in vitro* boar semen liquid preservation at 17°C. Meanwhile, 0.2 μM Lip-1 significantly inhibited lipid peroxidation, and improved acrosomal integrity, plasma membrane integrity, and MMP. Furthermore, 0.2 uM Lip-1 significantly enhanced sperm motility and kinetic parameters, and sperm quality in Era-treated sperm. This suggests that 0.2 μM Lip-1 may enhance the liquid preservation efficiency of pig semen at 17°C associated with alleviating ferroptosis. This finding offers theoretical and practical reference for the application of Lip-1 *in vitro* boar semen liquid preservation at 17°C. AI using semen stored at room temperature can provide a more accurate assessment of sperm quality. Consequently, our subsequent research will concentrate on evaluating the application of Lip-1 in enhancing the efficiency of room-temperature storage of pig semen within the pig farming industry.

## Data Availability

The original contributions presented in the study are included in the article/supplementary material, further inquiries can be directed to the corresponding author.
